# Multi-omics insights into triticale silage as a sustainable alternative to corn silage in heifer diets

**DOI:** 10.3389/fmicb.2026.1761287

**Published:** 2026-03-17

**Authors:** Yujie Niu, Chuying Wang, Yu Kuang, Xiaoxue Ma, Shanshan Nan, Peng Zhang, Qicheng Lu, Yayin Qi, Cunxi Nie, Yanyan Wu, Wenju Zhang

**Affiliations:** 1 Animal Nutrition and Feed Science, College of Animal Science and Technology, Shihezi University, Shihezi, China; 2 Bingtuan Key Laboratory for Efficient Utilization of Non-Grain Feed Resources, College of Animal Science and Technology, Shihezi University, Shihezi, China

**Keywords:** economic efficiency, fatty acid profile, growing heifers, metabolomics, rumen microbiome, triticale silage

## Abstract

**Background:**

Intensive ruminant production systems rely heavily on corn silage (CS) as a primary forage source; however, its resource-intensive cultivation and environmental constraints necessitate the development of sustainable alternatives.

**Methods:**

In a 90-day feeding trial, 24 growing heifers were assigned to diets in which CS was replaced by triticale silage (TS) at 0, 25, 50%, or 100% (DM basis). Growth performance, rumen fermentation, ruminal fatty acid (FA) profiles, and integrated rumen microbiome-metabolome interactions were evaluated.

**Results:**

A 25% substitution (TS25) as the optimal level, maintaining growth performance comparable to the control while achieving the lowest feed cost of gain. TS25 improved rumen fermentation (lower A: P and high total VFA), promoted more efficient nitrogen utilization (higher MCP with lower ammonia N), and enriched functionally relevant bacteria associated with fiber degradation and fermentation (e.g., *Ruminococcus*, *Prevotella*, and *Rikenellaceae_RC9_gut_group*). Consistently, TS inclusion shifted ruminal lipid metabolism, increasing UFA proportions and elevating PUFA (TS25 and TS50 increased PUFA by 15.2 and 23.7% vs. control), alongside metabolomic signals indicating upregulation of linoleic acid metabolism and aromatic amino acid biosynthesis pathways. In contrast, TS substitution ≥50% reduced DMI and ADG, impairing feed utilization.

**Conclusion:**

Partial replacement of CS with TS at 25% provides a practical, data-supported strategy to improve economic efficiency while maintaining productivity and promoting favorable rumen microbial-metabolic features. This feeding approach may be applicable in water-limited or double-cropping regions, where TS can enhance forage system sustainability without compromising heifer growth.

## Graphical abstract

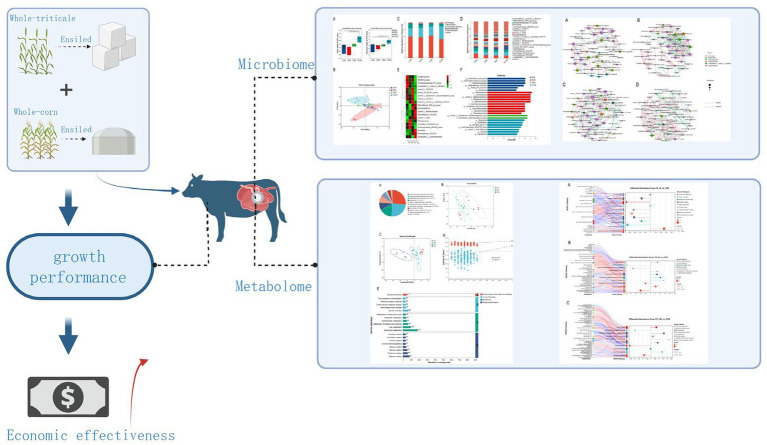

## 1 Introduction

In intensive ruminant production systems, high-quality roughage is essential for ensuring animal health and productivity. Corn silage (CS) is extensively used as a primary dietary component for ruminants owing to its high energy density, superior yield, and excellent palatability (Chibisa and Beauchemin, 2018). In arid regions such as Xinjiang, China, corn cultivation is constrained by water scarcity, which increases the costs of fertilization and irrigation. Although CS offers high energy density, prolonged high inclusion rates (>50% of diet DM) can disrupt ruminal homeostasis and increase acidosis risk due to elevated soluble carbohydrates (Penner and Oba, 2009; Lv et al., 2023). Triticale can mitigate these constraints by thriving under low-water conditions and enabling double-cropping systems (Arseniuk, 2015; Cui et al., 2025).

Triticale (×*Triticosecale Wittmack*), a wheat-rye hybrid crop well-adapted to cold and semi-arid conditions, provides a viable alternative forage to address these challenges (Arseniuk, 2015). Double-cropping rotation systems, such as triticale-corn, can increase land use efficiency and total annual forage yield. When sown after corn harvest, triticale can produce up to 26 t fresh biomass per hectare by the following May and may also reduce wind erosion (Cui et al., 2025). Ensiling harvested triticale is an economically viable method for preserving fresh forage. Triticale harvested at the milk stage contains approximately 10% water-soluble carbohydrates, which facilitate enhanced lactate production and rapid pH reduction during ensiling (Niu et al., 2025). Compared with CS, triticale silage (TS) has higher crude protein (CP) content (10% vs. 7% DM) (Niu et al., 2025; Santana et al., 2025) and neutral detergent fiber (NDF) concentration (59% vs. 41% DM), while maintaining higher *in vitro* NDF digestibility (58% vs. 52%) (National Academies of Sciences, Engineering, and Medicine, Division on Earth and Life Studies, Board on Agriculture and Natural Resources, Committee on Nutrient Requirements of Dairy Cattle, 2021). Starch concentrations in TS are highly variable (0.3–13.9% DM) and are predominantly influenced by harvest stage (Harper et al., 2017; Ashkvari et al., 2024). Moreover, triticale contains higher polyunsaturated fatty acid (PUFA) concentrations than corn, particularly *α*-linolenic acid (C18:3 *cis*-9,12,15; 11.17 vs. 1.10 g/100 g FA) (Khan et al., 2012) and linoleic acid (C18:2 *cis-*9,12; 40.02 vs. 31.36 g/100 g FA). These fatty acids (FA) may influence host metabolism through ruminal biohydrogenation processes.

Despite numerous advantages, TS also presents potential challenges due to its high fiber and low starch content. The high fiber content may increase ruminal fill and reduce dry matter intake (DMI), whereas the lower starch content may limit energy supply and thus restrict growth performance (Soest, 1994). Therefore, determining the optimal substitution level of TS for CS that maximizes nutritional and economic benefits while minimizing negative impacts on production remains a key research objective. Previous research on triticale in ruminants has primarily focused on nutritional evaluation and digestibility. Getachew et al. (2004) evaluated various roughages using *in vitro* gas production and found favorable ruminal fermentation characteristics for triticale. Krizsan et al. (2007) investigated how volatile compounds in silages affect cattle intake, establishing theoretical foundations for TS palatability research. Traditional approaches primarily relied on single-parameter evaluations (e.g., digestibility and performance measurements) and lacked comprehensive ruminal ecosystem analysis. Recently, researchers have increasingly adopted multi-omics approaches to investigate the effects of different feeds on ruminal microbial communities and metabolism. Harper et al. (2017) suggested that partial CS replacement with TS maintained milk yield but altered ruminal fermentation parameters in dairy cows. Recent research by Ashkvari et al. (2024) suggested that triticale hay substitution improved production performance in Holstein cows. Wang et al. (2023b) used multi-omics to reveal how key ruminal bacteria and metabolomes influence long-term lactation performance in dairy goats, offering new insights into feed-microbe-host interactions. However, these studies have methodological limitations and primarily focus on adult ruminants, with limited investigation in growing animals.

Therefore, this study aimed to evaluate the effects of replacing CS with TS at substitution levels of 0, 25, 50, and 100% on growth performance, rumen fermentation parameters, FA profiles, and integrated microbiome-metabolome in growing heifers, and quantifies economic implications. We hypothesized that low-level TS substitution maintains performance while improving rumen fermentation and unsaturated fatty acid (UFA) availability.

## 2 Materials and methods

### 2.1 Crop and silage

Triticale was cultivated at the experimental farm in Changji, Xinjiang, China (44°18′N, 86°27′E, elevation 370 m). A self-propelled forage harvester (JAGUAR 990–970, Claas, China) equipped with a kernel processor was used to chop the whole plant into a length of 2–3 cm. The chopped material was immediately ensiled in round bales, wrapped with six layers of 25 μm thick white polyethylene stretch film (Dingruifeng, China), and fermented under anaerobic conditions for 45 days. Subsequently, corn was harvested at the milk stage from the same site on September 13, 2023. The chopped corn was then transported to a bunker silo, where it was compacted in layers of 15–20 cm using a heavy tractor (LW500KV, XCMG Company, China) until achieving a target density of > 240 kg DM/m^3^. The silo was sealed with black-on-white polyethylene film and tire sidewalls to maintain anaerobic conditions for a minimum of 45 days. Before feeding, representative samples of TS and CS were collected to determine their chemical composition and FA profiles for diet formulation (Table 1).

**Table 1:** The nutritional composition and FA profile of the triticale and corn silage.

Items	CS	TS
Nutrient composition, % of DM		
DM, %	29.8	31.1
CP	7.30	11.2
Fat	3.56	4.18
NDF	53.2	61.1
ADF	29.7	37.1
ADL	5.14	9.56
Starch	25.3	11.7
Ash	4.68	6.55
pH	3.95	3.74
FA, g/100 g of total FA		
C12:0	0.365	0.204
C14:0	0.626	1.06
C16:0	27.9	15.9
C16:1 *cis*-9	0.253	0.237
C18:0	2.36	2.68
C18:1 *cis*-9	2.67	2.08
C18:2 *cis*-9,12	31.4	40.0
C18:3 *cis*-9,12,15	15.6	24.9

DM, dry matter; CP, crude protein; Fat, crude fat; NDF, neutral detergent fiber; ADF, acid detergent fiber; ADL, acid detergent lignin; CS, corn silage; TS, triticale silage.

### 2.2 Animals and diets

This study was conducted at the experimental cattle farm of Shihezi University in accordance with the Administration of Affairs Concerning Experimental Animals (Ministry of Science and Technology of China, revised 2004). The experimental protocol received approval from the Bioethics Committee of Shihezi University (Approval No. A2023-239).

Twenty-four crossbred heifers (Fleckvieh × Holstein F1; BW = 195 ± 10.5 kg; age = 6 ± 0.5 months) were randomly assigned using a number generator in Microsoft Excel to one of four treatments; each animal was treated as one experimental unit (*n* = 6 per treatment). The treatments consisted of total mixed rations (TMR) in which CS was replaced by TS at levels of 0% (CON), 25% (TS25), 50% (TS50), and 100% (TS100) on a dry matter (DM) basis. Prior to the start of the 90-day formal experiment, all heifers underwent a 14-day pre-experimental adaptation period. During this time, animals were gradually transitioned from the baseline diet to their respective experimental total mixed rations (CON, TS25, TS50, TS100) to allow for acclimation to the new feeding regimen and environmental conditions. Throughout this period, heifers were fed three times daily (0700, 1,400, and 2,100 h), and had ad libitum access to water. Health status was monitored daily to ensure all animals were healthy and consuming feed normally before data collection commenced. The experiment consisted of three consecutive 30-day periods. Each period comprised a 23-day adaptation phase followed by a 7-day sampling phase. This design allowed the animals to recover from the handling and sampling procedures of the previous period and to re-establish a stable baseline rumen environment before the next intensive sampling window, ensuring that the samples collected in each period were representative of a steady state. Diets were formulated to meet or exceed the nutrient requirements of growing heifers according to NRC (National Academies of Sciences, Engineering, and Medicine, Division on Earth and Life Studies, Board on Agriculture and Natural Resources, Committee on Nutrient Requirements of Dairy Cattle, 2021) recommendations, with nutrient composition varying according to the TS substitution level. The ingredient and nutrient composition of the experimental diets are presented in Table 2.

**Table 2:** Ingredient and chemical composition of the diets fed in the experiment.

Items	Treatments			
	CON	TS25	TS50	TS100
Ingredients, % of DM				
Alfalfa	14.5	14.5	14.5	14.5
Corn silage	58.0	43.5	29.0	0.00
Triticale silage	0.00	14.5	29.0	58.0
Wheat hay	7.25	7.25	7.25	7.25
Concentrate feed ^1^	20.3	20.3	20.3	20.3
Nutrient composition, % of DM				
ME, MJ/kg ^2^	11.2	11.1	10.9	10.8
CP	12.9	13.2	13.5	14.1
NDF	49.8	50.4	51.0	52.3
ADF	28.8	29.4	30.0	31.2
Fat	2.52	2.58	2.63	2.74
Ash	4.22	4.83	5.14	5.66
Starch	15.7	13.9	12.2	9.77

^1^ Formulated to provide (per kg of DM): ground corn 412 g, corn meal 193 g, cottonseed meal 110 g, brewer’s grains 50 g, wheat bran 100 g, DDGS 50 g, rice bran 50 g, molasses 20 g, dicalcium phosphate 10 g, magnesium oxide 2 g, and premix 3 g. The premix contained (per kg of DM): I 130 mg, Cu 400 mg, Zn 1950 mg, Se 30 mg, Co 60 mg, VA 25,000 IU, VD 42,000 IU, and VE 2,000 IU; ^2^ Estimated based on NRC (Nutrient requirements of dairy cattle: eighth revised edition, 2021), ME is calculated, while the other nutritional levels are based on measured values. CON, control; TS25, 25% TS in the silage portion; TS50, 50% TS in the silage portion; TS100, 100% TS in the silage portion.

### 2.3 Sampling

During each sampling period, daily feed offered and refusals were recorded to calculate feed intake. Total mixed ration (TMR) and refusal samples were collected at 3-day intervals to determine dry matter (DM) content and calculate DMI. Individual feed components, including roughages (CS, TS, alfalfa hay, cottonseed) and the concentrate mixture, were sampled weekly and stored at −20 °C for subsequent nutrient analysis. Composite samples of CS and TS were preserved at −80 °C for subsequent FA profile determination.

At the beginning and end of each experimental period, body weight was recorded to obtain initial body weight (IBW) and final body weight (FBW) for the calculation of average daily gain (ADG). Body weight gain (BWG) was calculated as the difference between FBW and IBW. ADG was calculated by dividing BWG by the number of experimental days. DMI was calculated as the difference between the DM of feed offered and DM of refusals. Feed to gain ratio (F:G) was calculated as the ratio of DMI to ADG. On the final day of each sampling period, prior to the morning feeding, rumen fluid samples were collected from each heifer via an oral stomach tube. To minimize saliva contamination, the initial 100 mL of collected fluid was discarded. Approximately 200 mL of rumen fluid was subsequently obtained and filtered through four layers of sterile cheesecloth. The filtered samples were divided into four aliquots for analysis of rumen fermentation parameters, FA profiles, bacterial composition, and metabolomic profiles, respectively.

### 2.4 Chemical composition and rumen fermentation parameter analysis

Total mixed ration and feed samples were oven-dried at 65 °C for 48 h, ground, and sieved through a 1-mm screen for chemical composition analysis. Crude protein (CP), crude fat, and ash were analyzed according to AOAC procedures (AOAC, 2005). Neutral detergent fiber (NDF) and acid detergent fiber (ADF) were determined using the methods described by Van Soest et al. (1991). Starch content was measured using a commercial assay kit (BC0700, Solarbio, Beijing, China) following the manufacturer’s instructions.

Rumen fluid pH was measured immediately upon collection using a portable pH meter (pH 3,110, WTW, Germany). Frozen samples were thawed at room temperature and centrifuged at 12,000 × *g* for 10 min at 4 °C. Supernatants were analyzed for ammonia nitrogen and volatile fatty acid (VFA) concentrations. For VFA analysis, metaphosphoric acid was added to supernatants, vortexed, and incubated for 2 h at room temperature prior to centrifugation at 13,500 × *g* for 10 min at 4 °C. Clarified supernatants were filtered through 0.22 μm syringe filters and analyzed by gas chromatography (Agilent Technologies, Santa Clara, CA, USA) as described by Wang et al. (2023a). Ammonia nitrogen concentrations were determined according to Verdouw et al. (1978). The concentration of microbial crude protein (MCP) in rumen fluid was determined using a commercial assay kit (TransGen Biotech Co., Beijing, China) according to the manufacturer’s instructions. Absorbance was measured with a fully automated microplate reader (Thermo Fisher Scientific, USA).

### 2.5 FA profile analysis

Fatty acid profiles of TS, CS, and rumen fluid were analyzed using a one-step methylation procedure adapted from Kramer et al. (1997). Freeze-dried samples (0.2–0.5 g) were transferred to capped glass tubes containing 200 μL nonadecanoic acid internal standard. Samples were treated with 8 mL of 0.5 mol/L sodium methoxide and heated at 50 °C for 15 min. After equilibration to ambient temperature, 8 mL of 2% methanolic sulfuric acid was added, and samples were incubated at 50 °C for 1 h. The reaction mixture was extracted with 4 mL n-hexane and 4 mL deionized water, vortexed, and centrifuged at 3,500 × g for 5 min. The upper organic layer was collected for gas chromatographic determination. Standard solutions containing 37 FA methyl esters (BYG8010, Solarbio, China) were serially diluted in *n*-hexane to generate five-point calibration curves. Standards were prepared at concentrations ranging from 1 to 1,000 μg/mL. Calibration parameters, including linearity, signal-to-noise ratios (S/N), and correlation coefficients (*R*^2^), are summarized in Supplementary Table S1.

Samples were analyzed using an Agilent 8,890 gas chromatograph coupled to an Agilent 7000D triple quadrupole mass spectrometer (GC-TQ/MS, Agilent Technologies, Santa Clara, CA, USA). Chromatographic separation was performed on an HP-INNOWax capillary column (30 m × 250 μm i.d. × 0.25 μm film thickness, Agilent Technologies, USA) using helium as carrier gas at a constant flow rate of 1.0 mL/min. Samples (1 μL) were injected in splitless mode with the injector temperature set at 260 °C. The oven temperature program was initiated at 80 °C (held for 0.25 min), ramped to 260 °C at 10 °C/min, and maintained at 260 °C for 3 min (total runtime: 21.25 min). The mass spectrometer was operated in electron ionization mode (70 eV) with ion source and quadrupole temperatures of 230 °C and 150 °C, respectively. Multiple reaction monitoring (MRM) mode was employed to enhance sensitivity and selectivity, with optimized transitions for each target analyte using specific precursor and product ion pairs. A solvent delay of 3 min was applied to prevent filament damage.

### 2.6 Rumen microbiome analysis

After thawing, 5 mL of rumen fluid was centrifuged at 13,000 × g for 15 min at 4 °C. The supernatant was discarded, and total bacterial DNA was extracted from the pellet using the DNeasy PowerSoil Kit (Qiagen, Germany) according to the manufacturer’s protocol. The extracted DNA was resuspended in 50 μL of sterile water, and its concentration and purity were quantified using a NanoDrop 2000 spectrophotometer (Thermo Fisher Scientific, Waltham, USA). The V3–V4 region of the 16S rRNA gene was amplified with 338F (5’-ACTCCTACGGGAGGCAGCAG-3′) and 805R (5’-GGACTACHVGGGTWTCTAAT-3′) (Frank et al., 2013). PCR products were confirmed by 2% agarose gel electrophoresis, and target amplicons were purified using the QIAquick Gel Extraction Kit (QIAGEN, Inc., Netherlands). Following quality control, libraries were normalized and sequenced on the Illumina MiSeq platform (Shanghai Personal Biotechnology Co., Ltd., Shanghai, China). Sequencing data underwent quality filtering using the Quantitative Insights into Microbial Ecology pipeline (QIIME 2) to eliminate low-quality reads. Amplicon Sequence Variants (ASVs) were delineated using the DADA2 algorithm (Callahan et al., 2016), with taxonomic classification performed by alignment against the Greengenes 2 database (McDonald et al., 2024). The ASV table was utilized to assess *α*-diversity and *β*-diversity. Bacterial community analysis was conducted using QIIME 2 for data processing and interpretation.

### 2.7 Rumen metabolomics analysis

After thawing, 1 mL of rumen fluid was centrifuged at 12,000 rpm for 10 min at 4 °C. The supernatant was combined with 3 mL of pre-chilled methanol to precipitate proteins, then incubated at −20 °C for 1 h. The mixture was subsequently centrifuged at 12,000 rpm for 15 min, and the resulting supernatant was filtered through a 0.22 μm membrane to yield the metabolite extract.

Metabolites were quantified using ultra-high performance liquid chromatography-mass spectrometry (UHPLC–MS, Agilent Technologies, Inc., Santa Clara, USA), with instrument settings adapted from Wang et al. (2023a). Briefly, sample aliquots (100 μL) were transferred to 2 mL centrifuge tubes and mixed with 400 μL extraction solution (acetonitrile\:methanol, 1:1 v/v, containing 0.02 mg/mL L-2-chlorophenylalanine as internal standard). Samples were vortexed for 30 s and subjected to ultrasonic extraction at 5 °C and 40 kHz for 30 min. Following incubation at −20 °C for 30 min, samples were centrifuged at 13,000 × *g* for 15 min at 4 °C. Supernatants were collected, dried under a nitrogen stream, and reconstituted in 100 μL reconstitution solution (acetonitrile: water, 1:1 v/v). Reconstituted samples were sonicated at 5 °C and 40 kHz for 5 min, centrifuged at 13,000 × *g* for 10 min at 4 °C, and supernatants were transferred to autosampler vials.

Metabolite analysis was performed using ultra-high performance liquid chromatography-mass spectrometry (UHPLC–MS, Agilent, USA). Chromatographic separation was achieved on an HSS T3 column (100 × 2.1 mm, 1.8 μm; Waters Corporation, Milford, MA, USA) with an injection volume of 3 μL. The mobile phase consisted of solvent A (95% water, 5% acetonitrile, 0.1% formic acid, v/v) and solvent B (47.5% acetonitrile, 47.5% isopropanol, 5% water, 0.1% formic acid, v/v/v). The flow rate was maintained at 0.40 mL/min with column temperature at 40 °C. Mass spectrometry was performed using a high-resolution mass spectrometer in both positive and negative ionization modes with a scan range of 70–1,050 m/z. Operating parameters included sheath gas flow (50 psi), auxiliary gas flow (13 psi), auxiliary gas heater temperature (425 °C), ion spray voltage (+3,500/−3,500 V), and ion transfer tube temperature (325 °C). Fragmentation was achieved using stepped collision energies of 20, 40, and 60 V in cyclic mode. Data acquisition employed a data-dependent mode with primary and secondary mass resolutions of 60,000 and 7,500, respectively.

Data were processed in Progenesis QI (Nonlinear Dynamics, Newcastle, UK) for peak picking, alignment, and normalization. Pooled quality control (QC) samples, prepared by mixing equal aliquots of all rumen extracts, were injected at the beginning of the run and every five samples to monitor signal stability. Features present in <80% of samples in at least one group, with signal-to-blank ratio <5, or with coefficient of variation (CV) > 25% in QC injections were removed. Isotopes and common adducts were deconvoluted, and one representative feature per metabolite was retained. Metabolites were annotated by accurate mass, retention time, and MS/MS matching to an in-house standard library and the HMDB and KEGG databases (mass tolerance ±10 ppm). Multivariate statistical analyses, including principal coordinates analysis (PCoA) and partial least squares discriminant analysis (PLS-DA). Differential metabolites were defined by FDR-adjusted *p* < 0.05 and log_2_FC ≥ 2.0. Metabolite identification and pathway mapping were conducted using the HMDB (accessed on March 15, 2024; https://hmdb.ca/pathways) and KEGG (accessed on March 20, 2024; http://www.kegg.jp/kegg/pathway.html) databases.

### 2.8 Statistical analysis

Initial data organization and summarization were performed using Microsoft Excel 20 (Microsoft Corp., USA). Statistical analyses were performed using SPSS Statistics 22.0 (IBM Corp., Armonk, NY, USA). For ruminal bacterial community analysis, *α*-diversity indices (Chao1 and Shannon) were calculated using QIIME 2 and compared among treatment groups using one-way analysis of variance (ANOVA) followed by Tukey’s multiple comparison test. For *β*-diversity analysis, PCoA based on Bray–Curtis dissimilarity matrices was performed to visualize community structure differences among treatments. Permutational multivariate analysis of variance (PERMANOVA) with 999 permutations was conducted to test the statistical significance of β-diversity separation among treatment groups. Differences in growth performance, ruminal fermentation parameters, rumen fluid FA profiles and bacterial community composition among treatments were assessed using Tukey’s multiple comparison test. Statistical significance was declared at *p* ≤ 0.05, and tendencies were considered when 0.05 < *p* ≤ 0.10.

## 3 Results

### 3.1 Chemical composition and FA profile of silages and diets

The nutrient composition and FA profiles of CS and TS are shown in Table 1, and the nutrient composition of the experimental diets is presented in Table 2. Compared to CS, TS had higher concentrations of DM, CP, crude fat, NDF, ADF, ADL, and ash. Conversely, CS showed a higher starch content. Regarding the FA profiles of the silages, TS had higher concentrations of C14:0, C18:2 *cis*-9,12, and C18:3 *cis*-9,12,15, reflecting a higher UFA concentration. In contrast, CS showed higher concentrations of C12:0, C16:0, C16:1 *cis*-9, C18:1 *cis*-9 and C18:0. Due to TS100 contained only TS as the silage, this diet had the highest concentrations of CP, NDF, ADF, ADL, and ash, and the lowest starch content among treatments (TS100 > TS50 > TS25 > CON; Table 2). The TS25 and TS50 diets showed intermediate nutrient composition, with greater crude fiber, lignin, crude protein, and ash concentrations than the CON, while maintaining starch levels between CON and TS100.

### 3.2 Growth performance and feed efficiency

IBW did not differ among treatments (*p* = 0.913), indicating comparable baseline conditions (Table 3). Dietary treatments significantly affected FBW, BWG, ADG, and DMI (*p* < 0.05). Compared to the CON group, FBW decreased by 4.7 and 8.6% in the TS50 and TS100 groups, respectively (*p* = 0.021). BWG decreased by 11.6 and 25.3% in the TS50 and TS100 groups relative to CON (*p* = 0.005), with corresponding ADG reductions of 11.5 and 25.1% (*p* = 0.005). Notably, growth performance in TS25 did not differ from the control (*p* > 0.05), indicating that a low TS substitution did not impair growth. DMI decreased by 6.8 and 15.1% in the TS50 and TS100 groups, respectively (*p* = 0.015). The F:G ratio was highest in the TS100 group (7.08; *p* = 0.024), a 13.6% increase compared to the CON group, indicating reduced feed efficiency at the 100% substitution level.

**Table 3:** Effects of partial replacement of corn silage (CS) with triticale silage (TS) on growth performance in heifers.

Items	Treatments				SEM	*p*-value
	CON	TS25	TS50	TS100		
IBW, kg	196	195	195	196	2.53	0.913
FBW, kg	298^a^	297^a^	284^b^	272^c^	3.69	0.021
BWG, kg	102^a^	102^a^	90.2^b^	76.2^c^	2.41	0.005
ADG, kg/d	1.13^a^	1.14^a^	1.02^b^	0.846^c^	0.026	0.005
DMI, kg/d	7.08^a^	6.98^a^	6.60^b^	6.10^c^	0.063	0.015
F:G	6.23^c^	6.15^c^	6.59^b^	7.08^a^	0.152	0.024

IBW, initial body weight; FBW, final body weight; BWG, body weight gain; ADG, average daily gain; DMI, dry matter intake; F:G, feed-to-gain ratio. CON, control; TS25, 25% TS in the silage portion; TS50, 50% TS in the silage portion; TS100, 100% TS in the silage portion. ^a − c^ within the row indicate significant differences among treatments (*P* < 0.05); SEM, standard error of means.

### 3.3 Rumen fermentation parameters

The rumen fermentation parameters are presented in Table 4. Ruminal pH showed no significant differences among groups (*p* = 0.645), but ammonia nitrogen and MCP concentrations were significantly affected by TS treatments (*p* < 0.05). Propionate concentration in TS25 was higher than in TS50 and TS100 (*p* < 0.05) but was not different from CON (*p* > 0.05). Butyrate and valerate concentrations decreased with increasing TS substitution level (*p* < 0.05). Total VFA concentration in TS50 and TS100 were lower than those in CON and TS25 (*p* = 0.038). The acetate-to-propionate ratio (A: P) was significantly lower in the TS25 group than in the CON group (*p* = 0.021), while fermentation efficiency increased significantly (*p* = 0.019).

**Table 4:** Effects of partial replacement of corn silage (CS) with triticale silage (TS) on rumen fermentation in heifers.

Items	Treatments				SEM	*P*-value
	CON	TS25	TS50	TS100		
pH	6.62	6.60	6.53	6.69	0.044	0.645
Ammonia N, mg/dL	9.32^a^	7.80^b^	8.25^b^	8.39^b^	0.139	< 0.001
MCP mg/dL	33.2^a^	35.5^a^	29.3^b^	27.1^b^	0.652	0.040
Acetate, mmol/L	43.5	45.8	44.8	43.1	0.763	0.208
Propionate, mmol/L	23.7^ab^	25.5^a^	22.3^b^	21.8^b^	0.408	0.024
Butyrate, mmol/L	6.91^ab^	7.15^a^	6.41^b^	6.21^b^	0.132	0.035
Isobutyrate, mmol/L	0.977	0.845	0.879	0.877	0.022	0.202
Valerate, mmol/L	0.745^a^	0.697^ab^	0.646^bc^	0.601^c^	0.017	0.011
Isovalerate, mmol/L	1.18^a^	0.911^b^	0.940^b^	0.924^b^	0.035	0.012
Total VFA, mmol/L^1^	78.5^a^	80.9^a^	75.0^b^	73.5^b^	1.22	0.038
A:P^2^	1.81^a^	1.74^b^	2.01^ab^	1.97^ab^	0.022	0.021
Fermentation efficiency^3^	0.782^b^	0.791^a^	0.784^b^	0.787^ab^	0.001	0.019

^1^VFA = volatile fatty acids; ^2^A:P ratio = acetate-to-propionate ratio; ^3^Fermentation efficiency = (0.622 × acetate+1.092 × propionate+1.56 × butyrate)/(acetate+propionate+2 × butyrate) (Abdl-Rahman et al., 2010). CON, control; TS25, 25% TS in the silage portion; TS50, 50% TS in the silage portion; TS100, 100% TS in the silage portion. ^a − c^ within the row indicate significant differences among treatments (*p* < 0.05); SEM, standard error of means.

### 3.4 Rumen fluid FA profile

TS inclusion altered the ruminal FA profile (Table 5). Specifically, increasing TS substitution reduced the proportion of saturated fatty acid (SFA), with a significant reduction in C16:0 concentration (*p* = 0.042), while C18:0 concentration shows an increasing trend (*p* = 0.079). The total SFA proportion significantly decreased from 77.7% in the CON to 73.9% in the TS100 group (*p* = 0.026). For monounsaturated fatty acids (MUFA), the proportions of C17:1 *cis*-10 (*p* = 0.013), C18:1 *trans*-9 (*p* = 0.051), and C18:1 *cis*-9 (*p* < 0.001), increased with higher TS inclusion. PUFAs including C18:2 *cis*-9,12 (*p* = 0.024), C18:3 *cis*-9,12,15 (*p* = 0.026), and C20:4 *cis*-5,8,11,14 (*p* < 0.001) showed significant increases. The proportion of total unsaturated fatty acids (total UFA) increased from 21.7% in the CON group to 25.5% in the TS100 group (*p* = 0.036), resulting in a lower SFA: UFA ratio (*p* = 0.064).

**Table 5:** Effects of partial replacement of CS with TS on rumen FA profiles in heifers.

Items	Treatments				SEM	*P*-value
g/100 g of FA	CON	TS25	TS50	TS100		
SFA						
C12:0	1.01	0.857	0.832	0.786	0.034	0.232
C13:0	0.644	0.573	0.577	0.606	0.014	0.246
C14:0	5.82	5.47	5.35	5.07	0.103	0.324
C15:0	3.43	3.21	3.11	2.88	0.102	0.472
C16:0	33.1^a^	31.2^ab^	29.8^b^	28.5^b^	0.617	0.042
C17:0	1.39	1.29	1.27	1.28	0.027	0.655
C18:0	17.9^b^	19.6^ab^	20.1^a^	20.9^a^	0.461	0.079
C20:0	3.28	3.07	3.22	3.18	0.068	0.188
C21:0	1.72	1.93	1.89	1.99	0.151	0.122
C22:0	3.52	3.16	3.53	3.11	0.086	0.234
C23:0	2.26	2.01	1.96	2.06	0.049	0.285
C24:0	3.58	3.30	3.47	3.65	0.073	0.706
MUFA						
C14:1 *cis*-9	0.973	0.811	0.832	0.882	0.061	0.130
C15:1 *cis*-10	0.896	1.07	1.13	1.00	0.029	0.254
C16:1 *cis*-9	1.16	1.18	1.14	1.14	0.024	0.931
C17:1 *cis*-10	1.21^c^	1.44^b^	1.43^b^	1.54^a^	0.058	0.013
C18:1 *trans*-9	1.23^b^	1.38^a^	1.44^a^	1.36^a^	0.032	0.051
C18:1 *cis*-9	3.44^c^	3.99^b^	4.27^ab^	4.61^a^	0.338	< 0.001
C20:1 *cis*-11	0.832	0.916	0.909	0.959	0.025	0.291
C22:1 *cis*-13	0.880	0.895	0.962	1.02	0.027	0.176
C24:1 *cis*-15	0.848	0.876	0.906	0.985	0.136	0.415
PUFA						
C18:2 *cis*-9,12	0.927^b^	1.11^a^	1.10^a^	1.20^a^	0.032	0.024
C18:2 *trans*-9,12	1.51	1.54	1.66	1.75	0.038	0.132
C18:3 *cis*-3,9,12	0.762	0.741	0.832	0.878	0.019	0.196
C18:3 *cis*-9,12,15	1.81^b^	2.23^ab^	2.35^a^	2.34^a^	0.023	0.026
C20:2 *cis*-11,14	0.870	1.06	0.943	1.00	0.026	0.328
C20:3 *cis*-8,11,14	0.636	0.583	0.577	0.606	0.017	0.219
C20:3 *cis*-11,14,17	0.904	1.12	0.984	1.04	0.047	0.138
C20:4 *cis*-5,8,11,14	0.327^c^	0.530^b^	0.500^b^	0.612^a^	0.031	< 0.001
C20:5 EPA	0.931	0.750	0.842	0.937	0.045	0.347
C22:2 *cis*-13,16	0.838	1.03	0.916	0.966	0.025	0.223
C22:6 DHA	0.775	0.766	0.882	0.740	0.032	0.124
Total SFA	77.7^a^	75.7^ab^	75.1^b^	73.9^c^	1.45	0.026
Total UFA	21.7^b^	24.1^ab^	24.6^ab^	25.5^a^	0.525	0.036
MUFA	11.4^c^	12.5^b^	13.0^a^	13.5^a^	0.375	0.013
PUFA	10.3^b^	11.4^ab^	11.6^ab^	12.1^a^	0.224	0.041
SFA: UFA	3.57^a^	3.15^ab^	3.04^ab^	2.89^b^	0.037	0.064

SFA, saturated fatty acid; UFA, unsaturated fatty acid; MUFA, monounsaturated fatty acid; PUFA, polyunsaturated fatty acids; SFA: UFA, saturated fatty acids to unsaturated fatty acid ratio; CLA, conjugated linoleic acid; EPA, eicosapentaenoic acid; DHA, docosahexaenoic acid; CON, control; TS25, 25% TS in the silage portion; TS50, 50% TS in the silage portion; TS100, 100% TS in the silage portion. ^a − c^ within the row indicate significant differences among treatments (*P* < 0.05); SEM, standard error of means.

### 3.5 Ruminal bacterial community composition

**Figure 1:**
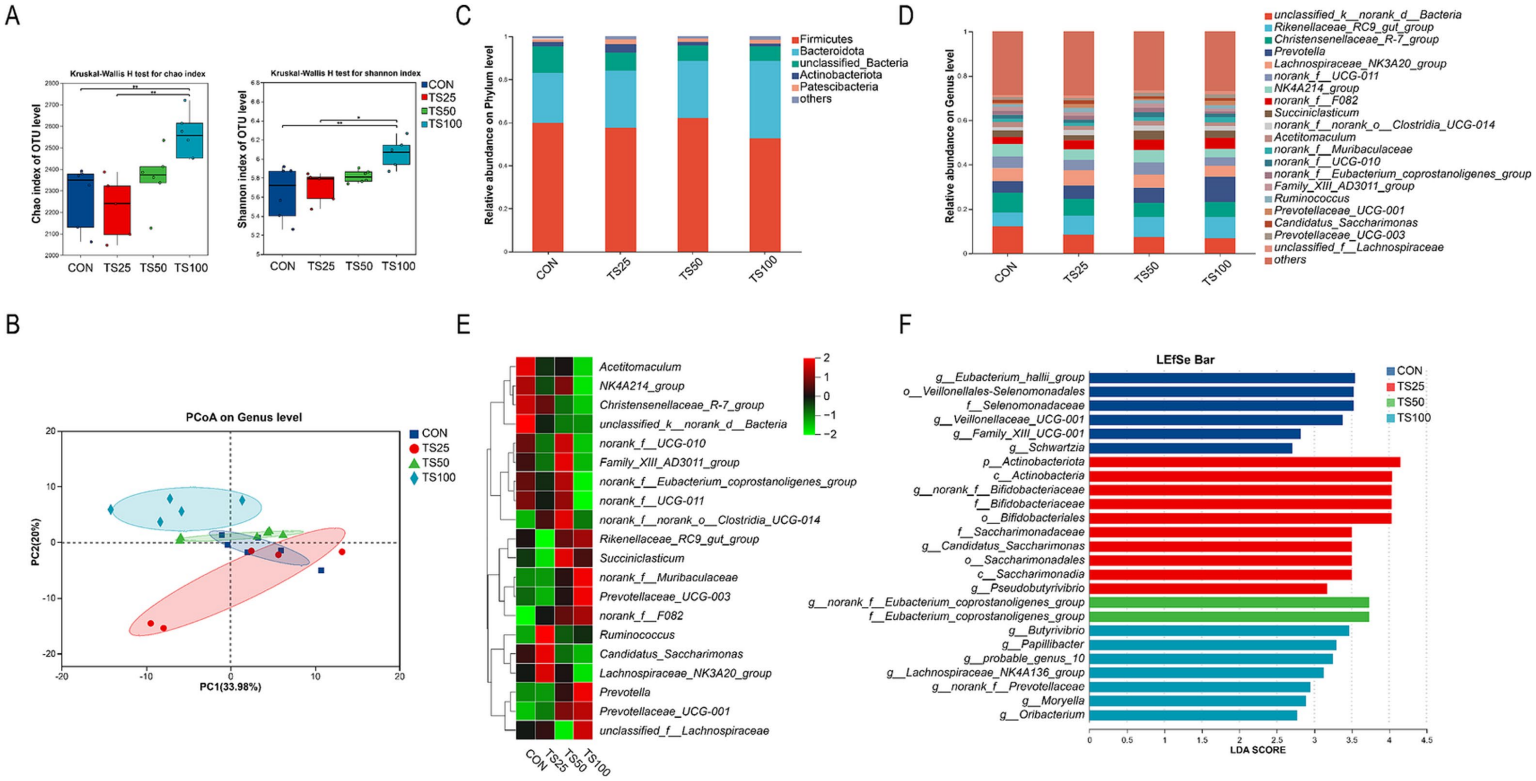
Rumen bacterial community structure and diversity analysis. **(A)** *α*-Diversity indices; **(B)** Principal coordinates analysis (PCoA) illustrating *β*-diversity; **(C)** Bacterial abundance composition at the phylum level; **(D)** Bacterial abundance composition at the genus level; **(E)** Cluster heatmap displaying bacterial abundance distribution; **(F)** LEfSe analysis identifying significantly differential taxonomic units (LDA score > 2.75). CON, control; TS25, 25% TS in the silage portion; TS50, 50% TS in the silage portion; TS100, 100% TS in the silage portion.

A total of 1,781,838 high-quality sequences were obtained from 24 rumen fluid samples, Good’s coverage above 98%, indicating sufficient sequencing depth for bacterial diversity assessment. As shown in Figure 1A, substituting TS for CS increased bacterial *α*-diversity (*p* < 0.05). For *β*-diversity, principal coordinates analysis (PCoA) showed clear separation among treatments (Figure 1B), indicating that TS substitution altered ruminal bacterial community structure.

**Figure 2:**
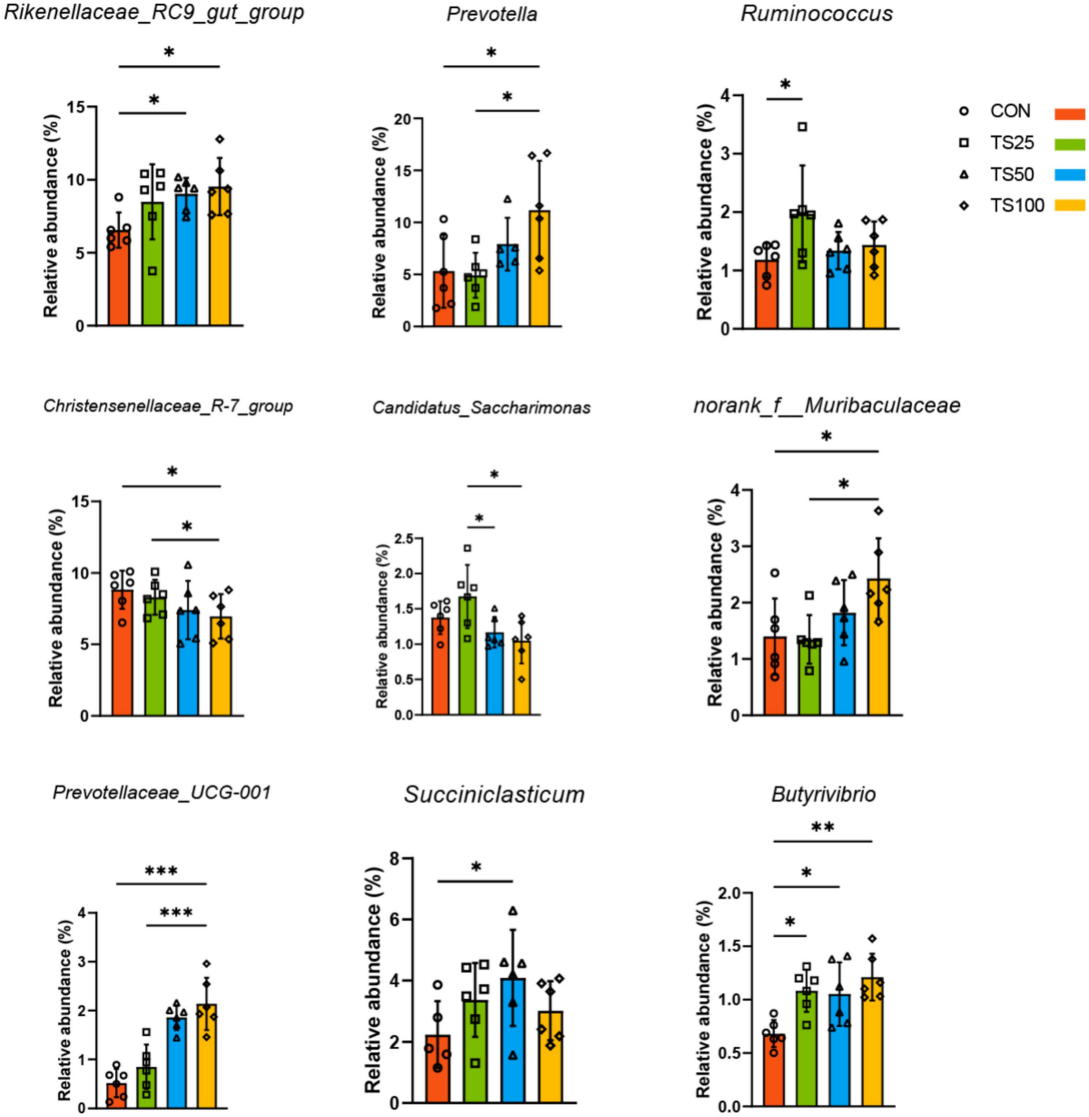
Bar chart of rumen differential bacterial abundance. The chart displays the relative abundance of significantly different bacterial taxa at the genus level across treatment groups. CON, control; TS25, 25% TS in the silage portion; TS50, 50% TS in the silage portion; TS100, 100% TS in the silage portion. * indicates significant difference (*p* < 0.05), ** and *** indicate extremely significant difference (*p* < 0.01).

At the phylum level (Figure 1C and Supplementary Table S2), Firmicutes (58.1%) and Bacteroidota (27.8%) were the dominant phyla across all treatments. Relative to the CON group, TS substitution significantly increased the abundance of Bacteroidota in the TS50 and TS100 groups (*p* < 0.05). At the genus level, TS substitution primarily affected taxa functionally associated with fiber degradation and fermentation end-product formation (Figures 1D,E, 2 and Supplementary Table S3). Fiber-associated genera *Prevotella* and *Rikenellaceae_RC9_gut_group* increased with higher TS levels (*p* < 0.05). Notably, *Ruminococcus* showed the highest abundance in TS25 compared to CON (*p* < 0.05), consistent with enhanced fiber utilization at moderate TS inclusion. *Succiniclasticum* was enriched by TS diets (*p* < 0.05), aligning with the more favorable rumen fermentation pattern observed in TS25. In addition, TS increased *Butyrivibrio* abundance (*p* < 0.05), a genus involved in lipid biohydrogenation, which is consistent with the TS-induced shift in ruminal fatty acid profiles. LEfSe analysis further identified group-specific bacterial signatures (LDA > 2.75; Figure 1F). CON was enriched in the *Veillonellaceae* lineage, TS25 in the *Bifidobacteriaceae* lineage, TS50 in the *Eubacterium coprostanoligenes* group, and TS100 in taxa including *Butyrivibrio*.

**Figure 3:**
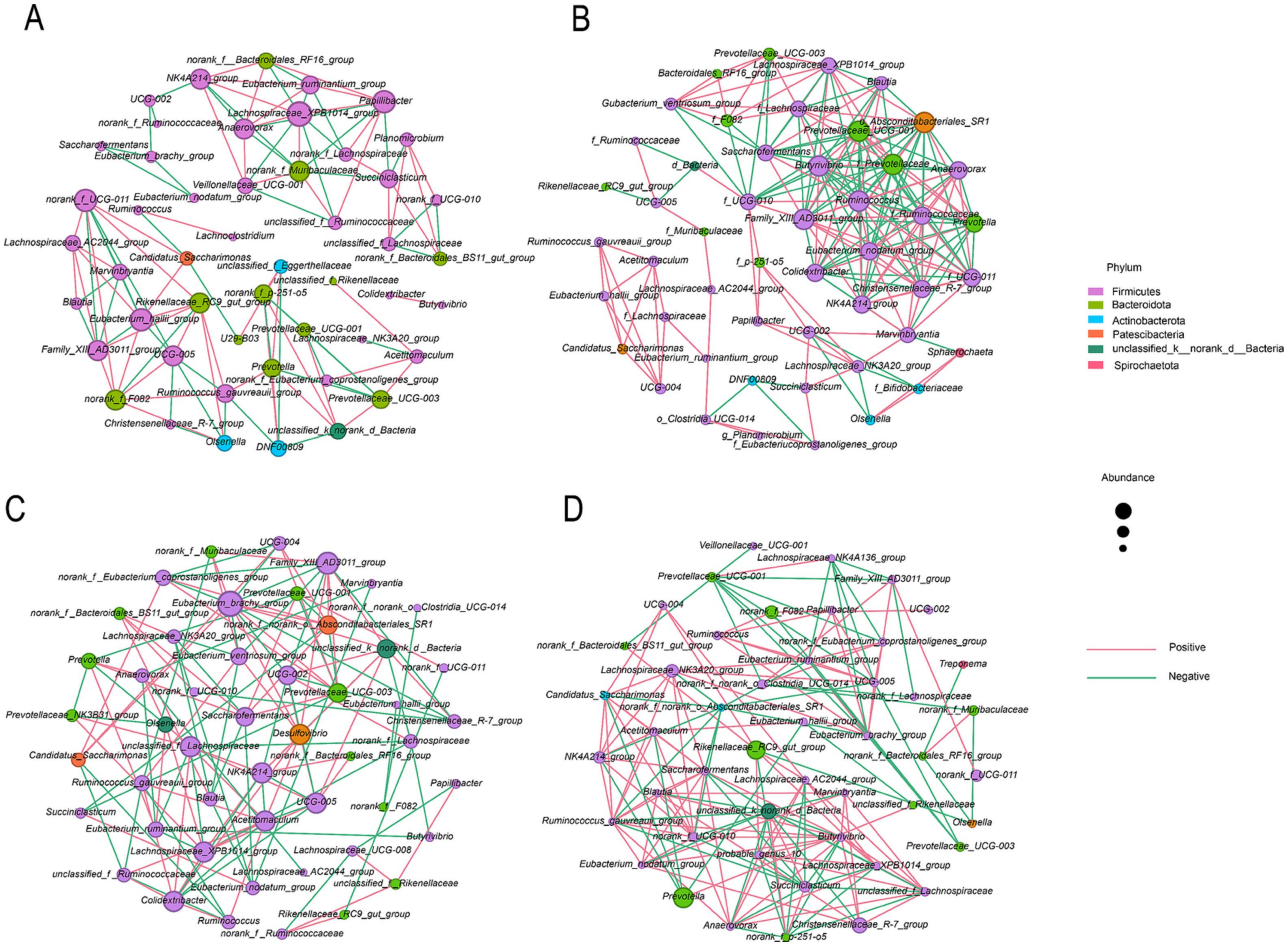
Bacterial co-occurrence network analysis. The network illustrates inter-taxa correlations among rumen bacterial genera across treatment groups: **(A)** CON (0% TS); **(B)** TS25 (25% TS); **(C)** TS50 (50% TS); **(D)** TS100 (100% TS). Nodes represent bacterial genera, with edge thickness and color indicating the strength and direction (positive or negative) of Spearman correlation coefficients.

TS substitution restructured rumen microbial co-occurrence networks (Figure 3 and Table 6). The TS25 group exhibited enhanced network complexity with 200 edges (+83.5% vs. CON’s 109), accompanied by a shift toward cooperative interactions (N*/*P = 0.770). In contrast, TS50 induced significant network destabilization characterized by renewed competitive dominance (N/P = 1.097; 52.31% negative edges), reduced modularity (clustering coefficient = 0.455, decrease 25.9% vs. CON), and increased path length (3.423, a 12% increase). The TS100 group established a cooperative equilibrium (N/P ratio = 0.677; 59.63% positive edges) with partially restored modularity (clustering coefficient = 0.593), although path length remained elevated (3.434). Node numbers remained stable across treatments (49 to 47 nodes), indicating network reorganization rather than community collapse.

**Table 6:** Topological properties of rumen microbial co-occurrence networks.

Metric	CON	TS25	TS50	TS100
Nodes	49	49	49	47
Edges	109	200	129	160
Negative (%)	50.9	43.5	52.3	40.4
Positive (%)	49.1	56.5	47.6	59.6
N/P ratio	1.04	0.77	1.10	0.677
Average clustering coefficient	0.614	0.662	0.455	0.593
Average path length	3.06	3.06	3.42	3.43

N/P ratio, the ratio of Negative (%) to Positive (%); CON, control; TS25, 25% TS in the silage portion; TS50, 50% TS in the silage portion; TS100, 100% TS in the silage portion.

### 3.6 Ruminal metabolomic profiles

**Figure 4:**
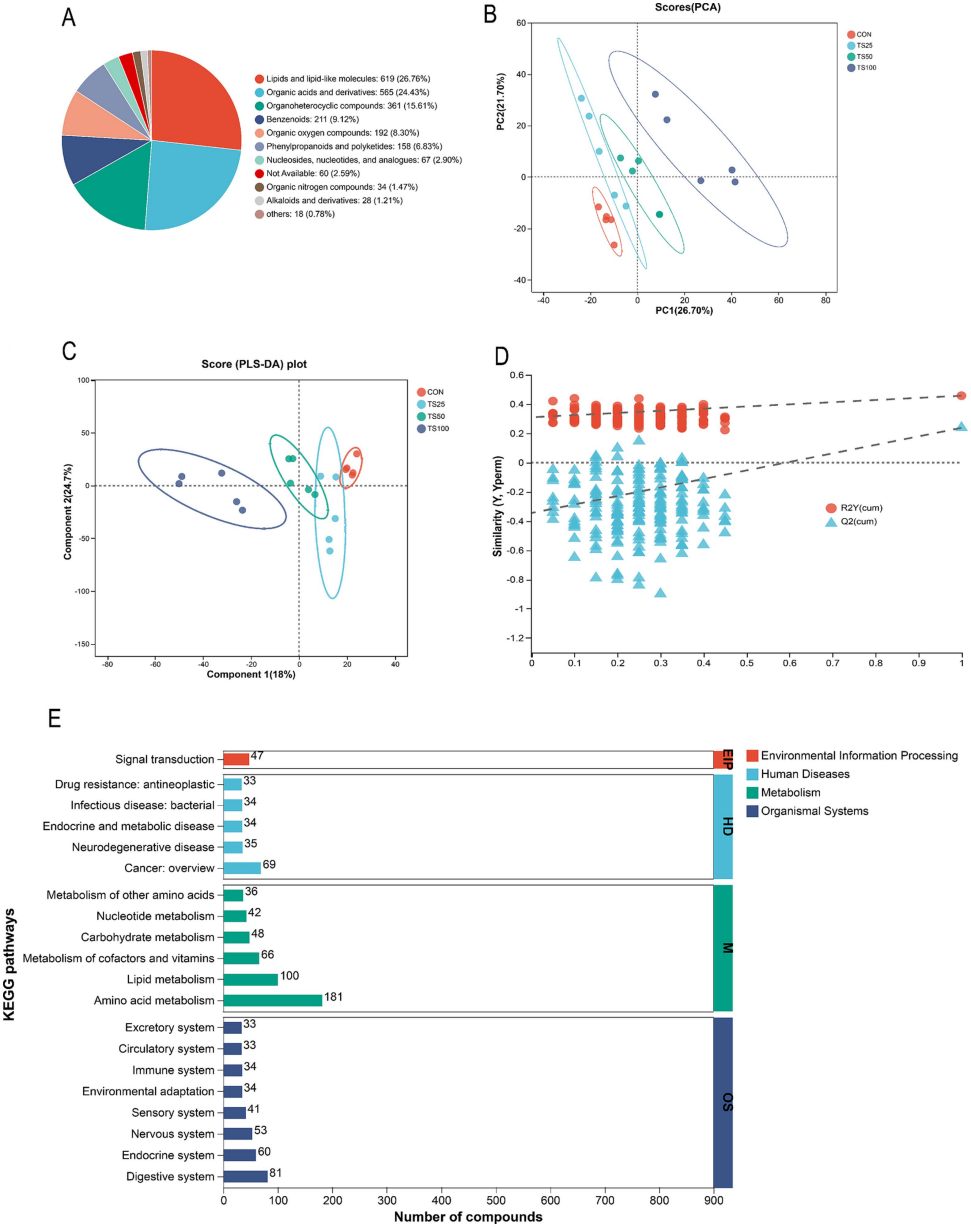
Rumen metabolomics of heifers diets with different proportions of TS replacing CS; **(A)** Chemical classification of the annotated metabolites in HMDB; **(B)** PCA score plots with different treatments; **(C)** Analysis of PLS-DA score plots with different treatments; **(D)** Analysis of PLS-DA model validation with different treatments; **(E)** KEGG pathway enrichment analysis of metabolites; CON (0% TS); TS25 (25% TS); TS50 (50% TS); TS100 (100% TS).

After QC, a total of 3,202 metabolites were identified across four treatment groups and categorized into 10 chemical classes. Lipids and lipid-like molecules (26.76%) constituted the largest proportion, followed by Organic acids and derivatives (24.43%) and Organoheterocyclic compounds (15.61%) (Figure 4A). To evaluate the impact of substituting TS for CS on rumen metabolites, PCA was performed. The first two principal components (PC1 and PC2) explained 48.4% of the total variance, with PC1 contributing 26.7% and PC2 contributing 21.7% (Figure 4B). The PCA scores plot revealed clear separation among the treatments, indicating distinct metabolic profiles for each group. Partial Least Squares Discriminant Analysis (PLS-DA) further substantiated these metabolic distinctions among the treatments (Figure 4C), suggesting that TS substitution for CS significantly altered rumen metabolite profiles. Validation of the PLS-DA model (Figure 4D) showed high predictive ability, indicating that the model was reliable and not overfitted.

For pathway analysis, ionization variants of the same metabolite were merged, yielding 1,060 unique metabolites that were mapped to KEGG pathways for enrichment analysis (Figure 4E). Among these, Metabolism emerged as the most active category, encompassing the largest number of compounds across its pathways. Within the Metabolism category, amino acid metabolism was the most prominent pathway, involving 181 compounds, followed by lipid metabolism (100 compounds), metabolism of cofactors and vitamins (66 compounds), carbohydrate metabolism (48 compounds), and nucleotide metabolism (42 compounds).

### 3.7 Ruminal differential metabolite profiling

**Figure 5:**
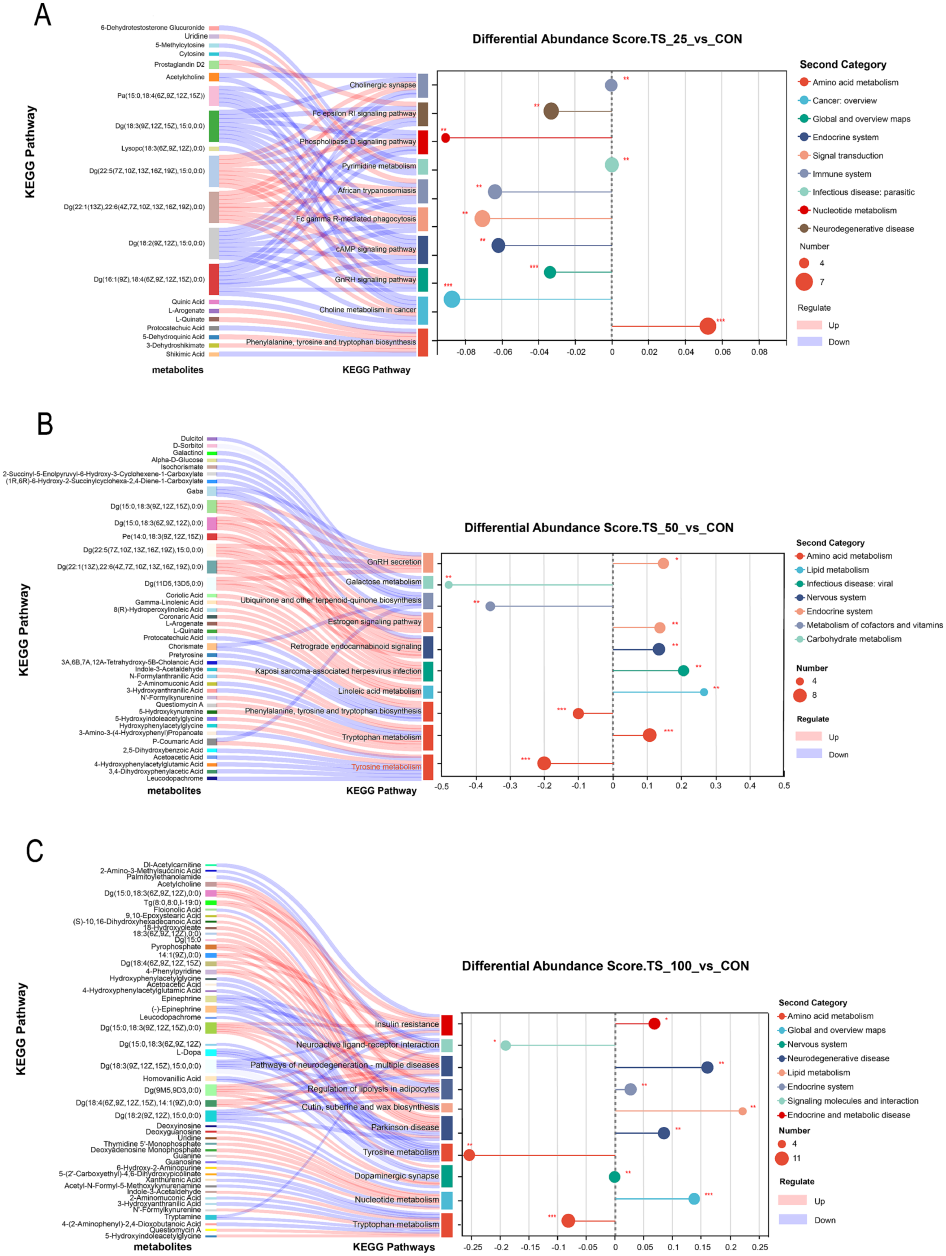
Differential metabolites and enriched pathways between TS treatments and CON. **(A)** TS25 vs. CON; **(B)** TS50 vs. CON; **(C)** TS100 vs. CON. CON, control; TS25, 25% TS in the silage portion; TS50, 50% TS in the silage portion; TS100, 100% TS in the silage portion.

The key differential metabolites were putatively annotated (MSI Level 2) based on high-accuracy MS/MS matching, providing a solid basis for mechanistic interpretation. As shown in Figure 5, the sankey diagram of differential metabolites and pathway enrichment score plot elucidated specific ruminal metabolic pathway alterations under TS treatments. Compared to the CON group, galactose metabolism-related metabolites such as galactinol, D-sorbitol, and dulcitol were significantly downregulated in the TS50 group. The phenylalanine, tyrosine, and tryptophan biosynthesis pathway related to amino acid metabolism was significantly upregulated in the TS25 group, with metabolites including L-arogenate, L-quinate, 5-dehydroquinic acid, and 3-dehydroshikimate being significantly upregulated. Lipid metabolism pathways (linoleic acid metabolism; cutin, suberine, and wax biosynthesis) were upregulated in TS50 and TS100, with corresponding increases in coriolic acid, gamma-linolenic acid, 8(R)-hydroperoxylinoleic acid, 9,10-epoxystearic acid, (S)-10,16-dihydroxyhexadecanoic acid, and 18-hydroxyoleate. Amino acid metabolism-related tyrosine metabolism and tryptophan metabolism were downregulated in the TS100 group. These findings suggest that TS substitution, particularly at high levels, substantially altered ruminal carbohydrate, amino acid, and lipid metabolism.

### 3.8 Economic analysis of triticale silage substitution

To evaluate the economic implications of substituting CS with TS, the feed cost per kilogram of body weight gain was calculated. The analysis utilized the observed growth performance data (Table 3) and diet compositions (Table 2). Market-representative costs were applied as shown in Table 7. The cost of each experimental diet was determined based on its ingredient composition. As shown in Table 8, the cost per ton of dietary DM decreased linearly as the proportion of TS increased, reflecting the lower cost of TS compared to CS. The economic analysis revealed that while the total daily feed cost progressively decreased with higher TS inclusion. The TS25 diet resulted in the lowest feed cost of gain at ¥10.33/kg. This represents a 4.0% cost saving per kg of gain compared to the CON diet (¥10.76/kg). The TS50 diet had a lower daily cost; its reduced ADG led to a slightly higher cost of gain (¥10.72/kg), comparable to the CON. The TS100 diet, despite being the cheapest per day, had the highest feed cost of gain (¥11.60/kg gain).

**Table 7:** Cost calculation for experimental diets.

Items	CON	TS25	TS50	TS100
Diet Cost (¥/ton DM)				
Alfalfa	260	260	260	260
Corn Silage	775	581	387	0
Triticale Silage	0	163	327	655
Wheat Hay	73.1	73.1	73.1	73.1
Concentrate	608	608	608	608
Total Diet Cost (¥/ton DM)	1,717	1,687	1,658	1,598

CON, control; TS25, 25% TS in the silage portion; TS50, 50% TS in the silage portion; TS100, 100% TS in the silage portion.

**Table 8:** Economic performance of heifers fed diets with varying levels of TS.

Items	CON	TS25	TS50	TS100
Performance data				
DMI (kg/d)	7.08	6.98	6.62	6.14
ADG (kg/d)	1.13	1.14	1.02	0.846
Economic calculation				
Total diet cost (¥/ton DM)	1,717	1,687	1,658	1,598
Total daily feed cost (¥/day)	12.2	11.8	10.9	9.75
Feed cost of gain (¥/kg gain)	10.8	10.3	10.7	11.6

CON, control; TS25, 25% TS in the silage portion; TS50, 50% TS in the silage portion; TS100, 100% TS in the silage portion.

## 4 Discussion

### 4.1 Impact of triticale substitution on energy balance and growth

The observed decline in growth performance (ADG, BWG, and F:G) and DMI at high TS substitution levels (TS50 and TS100) can be attributed to a metabolic energy deficit. Compared to CS, the TS treatments had higher fiber (61.12% vs. 53.23%) and lower starch content (11.67% vs. 25.31%), which created a less energy-dense diet, leading to physical rumen fill and slowed passage rate, thereby limiting feed intake and growth performance of heifers (Soest, 1994; Beauchemin and McGinn, 2011). This interpretation is supported by the metabolomics data. The significant downregulation of the “galactose metabolism” related to “carbohydrate metabolism” pathways in the high-TS groups indicates a primary substrate limitation. Carbohydrate metabolism pathway reconstruction revealed the substantial impact of altered fiber-starch ratios on ruminal fermentation patterns. Galactose metabolism-related metabolites (galactinol, D-sorbitol, dulcitol) were significantly downregulated in TS50 and TS100 groups, correlating with reduced total VFA concentrations (78.5 vs. 73.5 mmol/L) and decreased starch content (15.67% vs. 9.77%). This metabolic shift suggests that high-fiber diets limit readily fermentable carbohydrate availability, prompting ruminal microorganisms to alter their metabolic strategies. Ranathunga et al. (2019) reported similar metabolic reprogramming, whereby high-fiber diets maintain ruminal fermentation stability by restricting rapidly fermented substrate availability. Total VFA concentration, which represents the primary energy source for ruminants (Bannink et al., 2006), was significantly lower in the TS50 and TS100 groups than in CON (Table 4). This reduction is likely a combined consequence of both lower DMI and the lower starch content of the TS diets, which limits the availability of readily fermentable carbohydrates for microbial conversion into energy (Oba and Allen, 1999). Therefore, although TS provides higher CP content, the energy deficit created at high substitution levels appears to be the primary factor limiting animal growth.

### 4.2 Moderate substitution optimizes fermentation efficiency and microbiome stability

The 25% TS diet appeared to be the optimal substitution level, maintaining growth performance comparable to the CON group while inducing favorable shifts in rumen fermentation. The lower A: P ratio and higher fermentation efficiency in the TS25 group indicate a more favorable fermentation pattern, redirecting metabolic hydrogen towards the production of glucogenic propionate (Bergman, 1990). This metabolic shift is closely associated with the observed changes in the rumen microbiome. Substitution with TS reshaped the rumen microbial ecosystem, leading to increased *α*-diversity, which is generally indicative of a more resilient and functionally robust microbial community (Lozupone et al., 2012; Shade et al., 2012). Notably, the relative abundance of *Ruminococcus*, a cellulolytic genus (Ze et al., 2012), and *Succiniclasticum*, a specialist in converting succinate to propionate (van Gylswyk, 1995), peaked in the TS25 group. This suggests that a moderate increase in dietary fiber from 25% TS stimulated key fibrolytic and propionate-producing pathways without overwhelming ruminal digestive capacity, thereby improving energy extraction from the diet. Furthermore, the stable growth in the TS25 group was mirrored by a stable rumen metabolome, with amino acid and energy metabolite profiles largely indistinguishable from the CON group, indicating that this optimized rumen function compensated for the slight reduction in dietary starch. However, in the TS50 and TS100 groups, the severe deficit of readily fermentable starch ultimately overwhelmed this microbial compensatory capacity, leading to insufficient propionate and total VFA production, which directly contributed to the observed decline in growth performance. These findings provide a microbiological and metabolomic basis for the sustained performance at this substitution level and extend previous studies that focused primarily on production responses.

The observed phylum-level shift from Firmicutes towards Bacteroidota with increasing TS is consistent with response to diets with a higher fiber-to-starch ratio (de Menezes et al., 2011). This may also explain the propionate concentration in the TS25 group is similar to that in the CON group. At the genus level, the enrichment of fiber-degrading taxa such as *Prevotella* and *Rikenellaceae_RC9_gut_group* in the high-TS groups aligns with the increased fiber content of these diets. Interestingly, the relative abundance of *Christensenellaceae_R-7_group*, often associated with a lean phenotype and efficient fiber metabolism (Waters and Ley, 2019), decreased with TS substitution. This seemingly contradictory result may reflect a competitive disadvantage in the specific substrate environment created by TS or highlight functional redundancy within the fibrolytic microbial community. This might suggest that while both *Rikenellaceae* and *Christensenellaceae* are fibrolytic, they may specialize in different fiber fractions or thrive under different ruminal conditions, with the environment created by TS favoring the former. *Rikenellaceae_RC9_gut_group* contributes to acetate and propionate production in the rumen and plays a key role in maintaining propionate concentrations (Su et al., 2014). Previous studies indicate that both *Rikenellaceae_RC9_gut_group* and *Ruminococcus* are involved in cellulose degradation and protein fermentation (FitzGerald et al., 2015; Yang et al., 2020), which aligns with their observed enrichment in high TS substitution groups. LEfSe analysis further identified unique microbial biomarkers for each dietary regimen (e.g., *Veillonellaceae* in CON, *Bifidobacteriaceae* in TS25, *Butyrivibrio* in TS100), providing potential targets for future microbiome-based dietary interventions.

Co-occurrence network analysis revealed that TS substitution affected rumen microbial ecosystem stability. The TS25 group exhibited a more complex and stable network with enhanced synergistic relationships, a known feature of robust and efficient microbial ecosystems (Faust et al., 2012). Conversely, the TS50 network showed signs of destabilization, such as reduced clustering and increased negative correlations, marking a functional tipping point that preceded the decline in performance (Scheffer et al., 2009). The TS100 network reorganized into a distinct alternative steady state adapted to the high-fiber substrate (Zaneveld et al., 2017). Collectively, these results establish network topology as a sensitive indicator for predicting nonlinear physiological responses to dietary interventions (Gilbert et al., 2018).

### 4.3 TS modulates lipid biohydrogenation and amino acid metabolism pathways

Integration of metabolomics provided mechanistic insights into the functional consequences of the dietary and microbial shifts, particularly with respect to lipid metabolism. The higher concentration of PUFAs, specifically linoleic (C18:2) and *α*-linolenic (C18:3) acids, in TS served as a key driver for changes in rumen biohydrogenation. Metabolomic data revealed significant upregulation of the linoleic acid metabolism pathway, a finding corroborated by the rumen FA profiles (Table 5). The linoleic acid metabolism pathway was significantly upregulated in both TS50 and TS100 groups relative to CON, characterized by enrichment of key metabolites including Coriolic acid and Gamma-linolenic acid. These results align with observed ruminal FA profile changes, suggesting that elevated PUFA content in TS (C18:2: 40.02% vs. 31.36%; C18:3: 24.89% vs. 15.67%) enhanced microbial biohydrogenation processes. These findings corroborate Jenkins et al. (2008), demonstrating that PUFA biohydrogenation enhances ruminal fermentation efficiency and extend Shingfield et al. (2013) theoretical framework on dietary FA influences on ruminal metabolism. We observed an accumulation of the final biohydrogenation product stearic acid (C18:0) and key intermediates such as C18:1 isomers, alongside an overall increase in total UFA in the rumen fluid of TS-fed heifers. This suggests that TS not only supplies more PUFA precursors but also stimulates the microbial pathways responsible for their transformation (Lourenço et al., 2010; Shingfield et al., 2013). Notably, the relative abundance of *Butyrivibrio*, which converts C18 PUFA to trans FAs (Dewanckele et al., 2020), was elevated in the TS treatments (Figure 1E), paralleling the higher concentrations of trans FAs measured in these groups. Although the direct impact of these specific FA shifts on the observed growth performance requires further investigation, these intermediates are known to have potent biological activities, influencing host metabolism and inflammation (Bessa et al., 2000; Catala, 2017). The integrated analysis provides a mechanistic link between dietary FA supply and microbial metabolic output, a dimension that is often not addressed in traditional feed evaluation studies. In this study, the FA profiles are from rumen fluid. While an increase in ruminal PUFA and UFA is a necessary precursor for enriching these FAs in animal tissues, the extent of tissue deposition is influenced by subsequent intestinal biohydrogenation, absorption, and metabolic processes. Therefore, direct measurement of muscle or adipose tissue FAs in future studies is essential to confirm the translation of these ruminal changes into enhanced nutritional attributes of the final product.

Differential regulation of amino acid metabolism pathways provided additional insights into how TS may influence protein utilization. In the TS25 group, phenylalanine, tyrosine, and tryptophan biosynthesis pathways were significantly upregulated, with concurrent enrichment of key precursors including L-arogenate and L-quinate. This pattern indicates that moderate TS substitution enhanced *de novo* aromatic amino acid synthesis. Conversely, tyrosine and tryptophan metabolism pathways were downregulated in the TS100 group, suggesting the detrimental effects of excessive substitution on amino acid metabolic balance. These results align with amino acid metabolism patterns reported by Wang et al. (2023a) in goats, highlighting the critical role of nutritional balance in ruminal metabolic homeostasis. Coupled with the observed decrease in ruminal ammonia nitrogen concentrations across TS treatments (9.32 vs. 8.39 mg/dL), these findings suggest that TS may enhance nitrogen utilization through improved amino acid metabolic efficiency. These findings were corroborated by the MCP results. As the TS substitution level increased, the ammonia nitrogen exhibited a decline; however, MCP peaked in the TS25 group and decreased in the TS50 and TS100 groups. This may indicate that a moderate supply of degradable protein jointly promotes the efficient conversion of nitrogen into MCP, rather than being caused by reduced proteolytic activity (Bach et al., 2005). This aligns with the metabolomic results that the maintenance of relatively high propionate levels, enhanced activity of *Succiniclasticum* and *Ruminococcus*, and the upregulation of aromatic amino-acid biosynthesis, suggesting that the TS25 diet provides a synchronized supply of fermentable carbohydrates and degradable proteins. Such synchrony furnishes the necessary ATP and carbon skeletons for microbial protein synthesis (Clark et al., 1992). However, at higher TS substitution levels, the insufficiency of fermentable substrates leads to fermentation energy limitations (indicated by reduced VFA and ammonia nitrogen), thereby diminishing the capacity for microbial nitrogen assimilation. Consequently, MCP synthesis becomes restricted, consistent with the downregulation of amino acid metabolic pathways and the observed constraints in growth performance.

### 4.4 Economic and ecological trade-offs

The economic analysis supports the partial substitution of CS with TS. The TS25 group yielded the lowest feed cost of gain (¥10.33/kg gain), a 4.0% reduction relative to CON, while maintaining animal performance. This finding underscores that production efficiency, rather than diet cost alone, dictates profitability. Higher inclusion levels (e.g., TS100) were economically inefficient (¥11.60/kg gain) due to suppressed growth linked to negative metabolic effects. When combined with the land-use and environmental benefits of triticale as a winter crop, the 25% substitution level offers a practical strategy for improving both biological performance and economic efficiency in heifer production systems. However, the economic advantage reported here is based on prevailing spot prices. Feed commodity markets are volatile. While our analysis provides a strong proof-of-concept under the studied conditions, the magnitude of the economic benefit from TS substitution is price-dependent.

**Figure 6:**
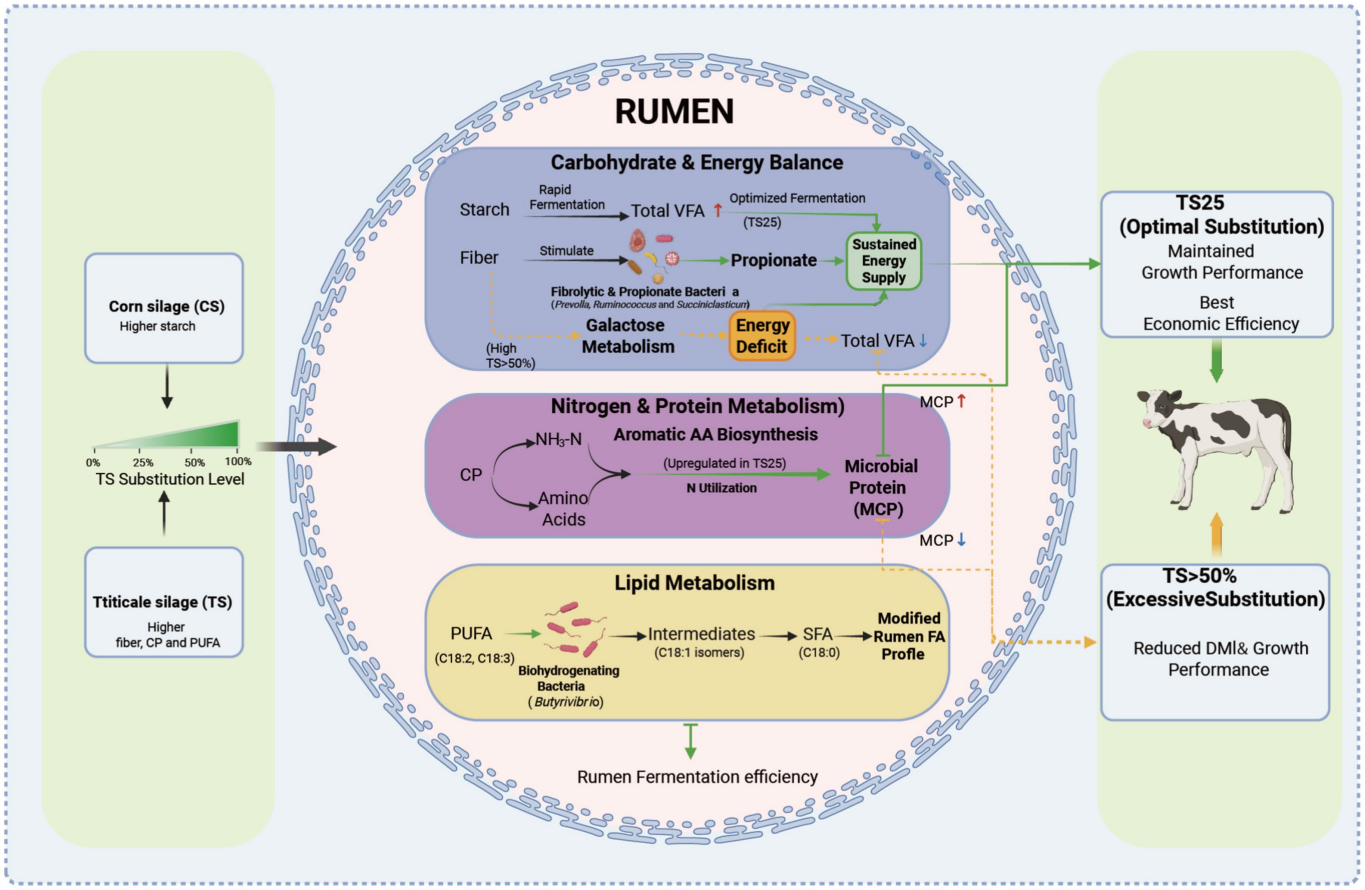
Integrated mechanism underlying the effects of substituting CS with TS on rumen function, microbial metabolism, fatty acid biohydrogenation, and animal performance in heifers. Green arrows indicate host-beneficial processes; yellow arrows indicate adverse effects; red arrows indicate increased or upregulated responses; blue arrows indicate decreased or downregulated responses.

The present findings align with and extend the existing literature. The negative impact of high-fiber forage on DMI and growth performance is well documented (Getachew et al., 2004; Krizsan et al., 2007). However, few studies have explored the effects of TS using a multi-layered, systems-biology approach. Previous research has identified TS as a viable forage alternative (Harper et al., 2017; Glamočlija et al., 2018; Ashkvari et al., 2024). Although the negative effects of high-fiber forage are known, this study provides a mechanistic explanation for the effects of TS substitution levels. This experiment was designed to evaluate the practical application and economic implications of TS; therefore, strict isocaloric balancing among treatment diets was not implemented. While isocaloric designs are useful for minimizing confounding factors, the FA profiles, amino acid compositions, and microbial indicators examined in this study are primarily influenced by the specific characteristics of forage type. Moreover, the difference in metabolizable energy between the TS25 group and the CON group was negligible (0.08%), and their growth performance was comparable, indicating that the absence of isocaloric adjustment did not compromise the validity of the conclusions. Nevertheless, in practical production systems using high proportions of TS, it may be advisable to moderately increase the concentrate proportion to compensate for the lower starch content of TS compared with CS. Multi-omics data, fermentation profiles, fatty acid metabolism, and growth performance together reveal a unified mechanism underlying the effects of TS substitution (Figure 6). A moderate substitution level (TS25) optimizes the synchronization between fermentable carbohydrates and degradable protein, thereby enhancing propionate production, stabilizing fibrolytic microbial networks, stimulating aromatic amino acid biosynthesis, and maximizing MCP. These coordinated microbial–metabolic interactions maintain ruminal energy supply and support growth, thereby explaining the superior biological and economic performance associated with TS25. In contrast, excessive substitution (TS50–TS100) increases dietary fiber and reduces starch to levels that limit fermentable substrate availability, suppress galactose metabolism, decrease total VFA production, alter lipid biohydrogenation pathways, and destabilize microbial co-occurrence networks. Collectively, these disruptions reduce ruminal energy yield and impair growth performance.

## 5 Conclusion

The 25% substitution level was identified as an optimal strategy, balancing high growth performance with superior economic efficiency. This was underpinned by improved rumen fermentation, an enriched fiber-degrading microbiota, and an enhanced FA profile with increased PUFAs. Conversely, higher substitution levels (≥50%) compromised animal performance due to energy deficit. This 25% substitution level also enhanced ruminal MCP synthesis while reducing the feed cost by 4.0%, supporting its use as a practical strategy to improve the economic efficiency of heifer production. Future work should quantify methane yield, rumen passage kinetics, and long-term carryover effects, and validate findings in lactating cows and other production systems.

## Data Availability

The datasets presented in this study can be found in online repositories. The names of the repository/repositories and accession number(s) can be found in the article/Supplementary material.

## Glossary

A:P: Acetate-to-propionate ratio
ADF: Acid detergent fiber
ADG: Average daily gain
ADL: Acid detergent lignin
AOAC: Association of Official Analytical Chemists
ASV: Amplicon Sequence Variant
BWG: Body weight gain
CON: Control (group)
CP: Crude protein
CS: Corn silage
DDGS: Dried Distillers Grains with Solubles
DHA: Docosahexaenoic acid
DM: Dry matter
DMI: Dry matter intake
DNA: Deoxyribonucleic acid
EPA: Eicosapentaenoic acid
F:G: Feed-to-gain ratio
FA: Fatty acid
FBW: Final body weight
GC-TQ/MS: Gas chromatograph-triple quadrupole mass spectrometer
HMDB: Human Metabolome Database
IBW: Initial body weight
KEGG: Kyoto Encyclopedia of Genes and Genomes
LDA: Linear discriminant analysis
LEfSe: Linear discriminant analysis Effect Size
MRM: Multiple reaction monitoring
MUFA: Monounsaturated fatty acid
NDF: Neutral detergent fiber
NRC: National Research Council
PCoA: Principal coordinates analysis (PCoA)
PCR: Polymerase Chain Reaction
PLS-DA: Partial least squares discriminant analysis
PUFA: Polyunsaturated fatty acid
QIIME: Quantitative Insights into Microbial Ecology
S/N: Signal-to-noise ratio
SEM: Standard error of means
SFA: Saturated fatty acid
TMR: Total mixed ration
TS: Triticale silage
UFA: Unsaturated fatty acid
UHPLC–MS: Ultra-high performance liquid chromatography-mass spectrometry
VFA: Volatile fatty acid
