# Supplementary material for: Multi-omics insights into triticale silage as a sustainable alternative to corn silage in heifer diets
Source: Front Microbiol. 2026 Mar 17;17:1761287. doi: 10.3389/fmicb.2026.1761287 (PMC13036108; doi:10.3389/fmicb.2026.1761287)
Supplement: Supplementary file 2 [file Table_2.DOCX]

| Table S2 microbial composition of rumen fluid at the phylum level % | | | | | | |
| --- | --- | --- | --- | --- | --- | --- |
| Phylum | Treatments | | |  | SEM | *P*-value |
|  | CON | TS25 | TS50 | TS100 |  |  |
| Firmicutes | 59.8 | 57.6 | 62.1 | 52.9 | 1.723 | 0.266 |
| Bacteroidota | 23.2b | 26.2ab | 26.3ab | 35.61a | 1.899 | 0.09 |
| norank_d__Bacteria | 12.37 | 8.61 | 7.30 | 6.96 | 1.034 | 0.217 |
| Actinobacteriota | 2.09b | 4.04a | 1.57b | 1.46b | 0.266 | 0.001 |
| Patescibacteria | 1.45b | 2.18a | 1.60ab | 1.48b | 0.113 | 0.088 |
|  |  |  |  |  |  |  |
