# Supplementary material for: Multi-omics insights into triticale silage as a sustainable alternative to corn silage in heifer diets
Source: Front Microbiol. 2026 Mar 17;17:1761287. doi: 10.3389/fmicb.2026.1761287 (PMC13036108; doi:10.3389/fmicb.2026.1761287)
Supplement: Supplementary file 3 [file Table_3.DOCX]

| Table S3 microbial composition of rumen fluid at the genus level % | | | | | | |
| --- | --- | --- | --- | --- | --- | --- |
| Genus | Treatments | | |  | SEM | P-value |
|  | CON | TS25 | TS50 | TS100 |  |  |
| *unclassified_k__norank_d__Bacteria* | 12.3 | 8.61 | 7.29 | 6.96 | 1.03 | 0.217 |
| *Rikenellaceae_RC9_gut_group* | 6.55b | 8.49ab | 9.03a | 9.53a | 0.427 | 0.08 |
| *Christensenellaceae_R-7_group* | 8.82a | 8.29a | 7.4ab | 6.95b | 0.349 | 0.061 |
| *Prevotella* | 5.31b | 5.11b | 7.91ab | 11.1a | 1.10 | 0.015 |
| *Lachnospiraceae_NK3A20_group* | 5.9 | 6.93 | 5.95 | 4.86 | 0.404 | 0.395 |
| *norank_f__UCG-011* | 5.41a | 4.88ab | 5.52a | 3.836b | 0.277 | 0.1 |
| *NK4A214_group* | 5.66 | 4.62 | 5.42 | 3.87 | 0.303 | 0.125 |
| *norank_f__F082* | 3.2 | 4.24 | 4.6 | 4.91 | 0.301 | 0.187 |
| *Succiniclasticum* | 2.23b | 3.36ab | 4.08a | 3.01ab | 0.420 | 0.042 |
| *norank_f__norank_o__Clostridia_UCG-014* | 1.82 | 2.19 | 2.46 | 1.92 | 0.111 | 0.153 |
| *Acetitomaculum* | 2.22 | 1.86 | 1.91 | 1.61 | 0.106 | 0.217 |
| *norank_f__Muribaculaceae* | 1.4b | 1.34b | 1.82ab | 2.42a | 0.211 | 0.025 |
| *norank_f__UCG-010* | 1.81 | 1.62 | 1.91 | 1.52 | 0.078 | 0.276 |
| *Eubacterium_coprostanoligenes_group* | 1.89a | 1.64ab | 2.08a | 1.16b | 0.119 | 0.022 |
| *Family_XIII_AD3011_group* | 1.75ab | 1.46ab | 2.10a | 1.31b | 0.121 | 0.084 |
| *Ruminococcus* | 1.18b | 1.97a | 1.34ab | 1.43ab | 0.118 | 0.114 |
| *Prevotellaceae_UCG-001* | 0.516b | 0.850b | 1.85ab | 2.14a | 0.276 | 0.097 |
| *Candidatus_Saccharimonas* | 1.37ab | 1.67a | 1.16b | 1.04b | 0.080 | 0.026 |
| *Prevotellaceae_UCG-003* | 1.12 | 0.979 | 1.31 | 1.72 | 0.123 | 0.164 |
| *unclassified_f__Lachnospiraceae* | 1.26 | 1.28 | 1.12 | 1.38 | 0.062 | 0.562 |
|  |  |  |  |  |  |  |
